# The grand challenge at the frontiers of neurotechnology and its emerging clinical applications

**DOI:** 10.3389/fneur.2024.1314477

**Published:** 2024-01-17

**Authors:** Roongroj Bhidayasiri

**Affiliations:** ^1^Department of Medicine, Faculty of Medicine, Chulalongkorn Centre of Excellence for Parkinson's Disease and Related Disorders, Chulalongkorn University, King Chulalongkorn Memorial Hospital, Thai Red Cross Society, Bangkok, Thailand; ^2^The Academy of Science, The Royal Society of Thailand, Bangkok, Thailand

**Keywords:** health technology, neurotechnology, neuroscience, neurological disorders, artificial intelligence

## Introduction

When I commenced my medical training, I could never have imagined the huge advances that would be made in the intervening years in the use of technology across all fields of medicine, and importantly the great opportunities it offers today to improve our understanding of diseases, their diagnosis, and the provision of care for patients. The evolution of health technologies, particularly in the last few decades, has been rapid and wide-ranging, and their potential is impressive. These new health technologies come in multiple different forms, many of which are already being integrated into our healthcare infrastructures – digital health apps, telemedicine, remote monitoring, and artificial intelligence (AI)-driven diagnostic tools. In addition, many countries are actively “digitizing” their health and social care systems.

## The technology revolution and its impact on neurological practice

The field of neurology is no exception, and in fact neurotechnologies have been described as the “next technology frontier” by the IEEE (Institute of Electrical and Electronics Engineers), the world's largest technical professional organization dedicated to advancing technology for the benefit of humanity (1). Neurotechnology describes the field of science and engineering in which the nervous system is interfaced with technical devices (2). Neurotechnologies can provide insights into brain or nervous system activity, or can influence brain or nervous system function (1). Essentially, neurotechnologies have the potential to help neuroscientists gather information that might help uncover some of the secrets of the biology underlying the normal and pathological functioning of the human brain – arguably the most complex and least understood organ of the human body – as well as delivering practical therapeutic or rehabilitative solutions in the clinical care of neurological disorders to help ease the personal and socioeconomic burden of these conditions (1, 3). Adopting a technology-based approach can also have benefits for research, allowing the use of more sensitive endpoints that will accelerate data gathering and evidence generation in clinical trials.

Some neurotechnology techniques, such as electroencephalograms (EEG) and magnetic resonance imaging (MRI), can be used to record and visualize brain activity. Neuromodulation describes the type of neurotechnology used in deep brain stimulation (DBS) for reducing tremors and other symptoms of Parkinson's disease, and in spinal cord stimulation for treating chronic pain (2). Other types of neurotechnology aim to replace or restore a patient's sensory, motor, or cognitive functions, one example being the use of cochlear implants to restore hearing in people with profound hearing loss (4). Many of these examples of neurotechnology are now well-established treatments used in routine clinical practice with some receiving approval by recognized regulatory authorities. DBS, for example, was approved by the US Food and Drug Administration in 1997 to treat essential tremor, in 2002 for Parkinson's disease, in 2003 for dystonia, and more recently in 2009 for obsessive-compulsive disorder (5).

These are just some examples of technologies that have been shown to yield positive clinical impacts in the management of patients with neurological disorders. However, as the field of neurotechnology is rapidly expanding, it is not possible to review all the available and investigational technologies in this editorial. What is clear is that these advances in neurotechnology are not something that will only impact us far into the future but are in fact happening ‘here and now' in our own neurology practices, hospitals, and even in patients' own homes. However, the exponential rise in the complexity of neurotechnologies and the speed of their development risks a potential disconnect between the developers who make them and clinicians who implement them. Developers will have a detailed knowledge of the particular technology but may lack a deep clinical understanding of the underlying disorder, the usual treatment pathway, the clinician's objectives and, critically, the patient's needs. On the other hand, while some new technologies may be easily available to them, clinicians may lack an understanding of how to apply them in the clinical setting and, importantly, how to integrate them into their usual healthcare delivery pathways. To make any new neurotechnology truly patient-centered requires insights from, and collaboration between, all stakeholders including patients themselves – from the initial development stage onwards (6).

Research and development of neurotechnologies continues to progress at a rapid rate (7–9) and it is likely that in the future we will see the further development and deployment of more sophisticated brain–computer interfaces (BCI) and robotic devices, advances in materials and technological methods, and the identification of new technologies that have the potential to help those with what used to be considered as irreversible neurological outcomes to regain their functional capacity and quality of life. In 2022, the prospective, open-label, non-randomized Brain Gate feasibility study, the largest and longest-running clinical trial of an implanted BCI, reported positive safety results in patients with quadriparesis from spinal cord injury, brainstem stroke, or motor neuron disease (10), paving the way for further clinical research. More recently, Elon Musk's BCI start-up company, Neuralink, has reported the development a novel BCI platform (11) and has now begun recruiting subjects with paralysis for its first human trial. While these technologies are very much at the experimental stage and evidence of their efficacy, safety and utility remains to be fully established, the possibilities they offer are exciting to contemplate.

## Ensuring effective governance and regulation of neurotechnology

The convergence of neurotechnologies with other emerging technologies, such as AI, is making their impact more unpredictable, disruptive and complex. As technologies intertwine and affect one another, they become harder to regulate, and comprehending and anticipating their long-term effects – both positive and negative – becomes increasingly elusive. While the curiosity and excitement about the promise of neurotechnologies should be embraced and encouraged, their limitations should also be recognized, as well as the valid concerns about governance, regulation, potential risks for patients (particularly with invasive technologies) and, importantly, considerations of ethics and human rights (12–14). Many neurotechnologies are under investigation but the pathway from research and development to clinical practice is often a protracted one that needs a collaborative effort to overcome the many technological, clinical, ethical, legal, and commercial challenges (15). As highlighted in a recent report from the United Nations Educational, Scientific and Cultural Organization (UNESCO), neurotechnologies have the potential to decode and alter perception, behavior, emotion, cognition and memory, which has major ethical implications in terms of mental privacy and modification of identity, beliefs and desires – our “humanness” – leading to a call for specialist “neuro-rights,” which would encompass the concepts of mental privacy (which proposes that we should have control over access to our neural data and to the information about our mental processes and states that can be obtained by analyzing it) and cognitive liberty (the freedom of an individual to control their own mental processes, cognition, and consciousness) (12, 14, 16, 17). Robust governance frameworks need to be in place to safeguard personal data privacy, particularly when private companies may be collating and commercializing these data for marketing and consumer engagement purposes. As these new neurotechnologies have the capacity to both read from and write into the brain, another real possibility that will require stringent governance policies is the concept of “brain hacking,” namely the manipulation, or even the weaponizing, of people and their behaviors, thoughts and feelings (18). While these uses are not yet a reality, they are a future risk, so it is important that the ethical and regulatory frameworks are discussed, agreed on, and implemented proactively to ensure safeguards are in place to prevent misuse when the time comes (18, 19).

Another important issue is the blurred line between what constitutes medical and what constitutes consumer neurotechnology (20). How do we ensure there is no crossover between sectors to ensure people's safety? Neurotechnology has been approved and used in the management of neurological disorders for decades but there is now rapid development in the consumer sector too and the use of neurodata for personal wellbeing, sports, marketing, and even workforce monitoring. Strict regulations are needed for development and testing, otherwise there may be a risk of generating inaccurate data and bias, potentially leading to discrimination and other harms (21).

Recognizing the need for responsible innovation in neurotechnology, the Organization for Economic Co-operation and Development (OECD) has developed the first international standard in this domain, which aims to guide governments and innovators to anticipate and address the ethical, legal and social challenges raised by novel neurotechnologies while continuing to promote innovation in the field. The OECD has identified, five possible systemic changes that could help speed up neurotechnology developments to meet pressing health challenges and societal needs while also ensuring this is undertaken responsibly and that the necessary safeguards are in place (22): (1) responsible research (this encourages consideration of ethical, legal and social issues ([ELSI] and collaboration between all stakeholders, including patients, patient organizations and funders, throughout the development process), (2) anticipatory governance (as discussed earlier, proactive consideration of ELSI so that frameworks can be put in place in good time), (3) open innovation (in light of the investment risks and high failure rates of clinical trials, neurotechnology companies could take an open innovation approach in which public and private stakeholders collaborate, invest, and share assets), (4) avoiding neuro-hype (controlling unproven claims and myths about neurotechnology and being realistic about what it can achieve by means of evidence-based policies and guidelines for its responsible development and use), and (5) access and equity (addressing socioeconomic questions and ensuring access to innovation in resource-limited countries. We need to consider all these factors as we move forward with neurotechnology to ensure that we reap its considerable benefits while minimizing any potential risks.

## What does the rise of neurotechnology mean for the future of neurology?

Neurotechnology already has the potential to alter the brain's chemistry and future developments predict that it will soon be able to create new neural connections between different parts of the nervous system (23). This will have huge therapeutic benefits for people with a range of neurological conditions, for example, stroke, chronic pain, paralysis, or psychological disorders, including anxiety and post-traumatic stress disorder. With today's neurotechnology, we are already able to use brain stimulation to help restore damaged memories in the brain (24, 25). While we need to be mindful of the OECD's recommendation to avoid “neuro-hype” and to be realistic about what is possible, within a decade, it is likely that commercial implants for memory stimulation will be available, and within 20 years, it is likely that neurotechnology will make it possible to manipulate memories, and even delete negative or traumatic ones. Significant developments are also expected to occur in the BCI and robotics sectors, for example transforming the lives of patients suffering from limb loss by allowing them to intuitively control a robotic arm.

Within the next few decades, and with the correct safeguards in place, it is likely that the rapid progress in neurotechnology will have a significant positive impact on many people's lives, not only in the field of neurology in terms of diagnosis and management, but also for individuals and wider society as a whole. Insights into the pathogenesis of diseases will facilitate implementation of preventive strategies as well as targeted treatments, allowing people to live longer, healthier, and more comfortable lives. In addition, neurotechnology will provide the possibility for the introduction of life-changing assisted technologies in both the home and the workplace. Achieving these ambitious objectives will require multiple stakeholders to engage in the responsible development and implementation of these promising technologies to ensure that the benefits for our society are fully realized.

A critical point that will need to be considered when developing these neurotechnologies is that they require “ground truth,” namely information that is known, through direct observation and measurement, to be real or true, to train and validate them. This can be complex to generate in the field of neurology where there is often unclear pathology, a range of diagnostic criteria, and observational or subjective data of varying quality, but is a challenge that will need to be overcome.

## Introducing *Frontiers of Neurology's* new “neurotechnology” section

Accepting these diverse challenges, it is clear that we are at an exciting point in translational neuroscience research. As the neurotechnology field grows and both scientific and public interest starts to expand, it is appropriate that there is a dedicated platform that allows researchers and others with an interest in the field to contribute to the overall scientific effort and communicate their own scientific expertise. A multi-faceted approach, bringing together basic, clinical, and technological research is required. We are therefore pleased to introduce a new “Neurotechnology” section of *Frontiers in Neurology* which aims to publish high-quality, fundamental and applied research, innovations, and potential clinical applications in any area of neuroscience where technological advancements have been employed. This interdisciplinary forum welcomes a wide range of articles related to neurotechnology. Potential topic areas include neuromodulation with technologies that use neural interfaces to record and/or stimulate nervous system structures, for example wearable or non-wearable devices, DBS, transcranial magnetic/direct current stimulation, focused ultrasound, or spinal/peripheral stimulation; neuro-prostheses which aim to restore lost motor, sensory or cognitive function, for example implanted technologies, robotic arms, or technologies to promote rehabilitation; and BCIs, for example cortical interfaces, robotic devices and hybrid systems. We are also interested in articles that fall outside of these three main areas, for example different imaging modalities of brain tasks, programmed pharmacology, gamification, brain training, and the new challenges that these technologies present, including ethics and legal implications. We welcome your insights.

Neurotechnologies can be used in various disciplines within the field of neuroscience, so we are mindful that some articles in this section may overlap with topics in other sections of *Frontiers of Neurology*. We see this as a valuable opportunity to highlight and integrate information about these technologies across the different areas of neuroscience, thereby improving interdisciplinary collaboration.

We hope that this dedicated journal section on neurotechnology will serve as a platform to share information on advances in these technologies, their evaluation in the diagnosis and management of various neurological disorders, and ultimately their potential for integration into routine clinical practice to benefit our patients and society as a whole.

## Author contributions

RB: Conceptualization, Resources, Validation, Visualization, Writing–original draft, Writing–review & editing.
